# Supplementary material for: Investigation on ABCC6-Deficient Human Hepatocytes Generated by CRISPR–Cas9 Genome Editing
Source: Cells. 2025 Apr 11;14(8):576. doi: 10.3390/cells14080576 (PMC12025709; doi:10.3390/cells14080576)
Supplement: Supplementary file 1 [file cells-14-00576-s001.zip › cells-3528531-supplementary.pdf]

cells

MDPI

Supplementary materials

Investigation on ABCC6-Deficient Human Hepatocytes Generated by CRISPR Cas9 Genome Editing

Ricarda Plümers *, Svenja Jelinek, Christopher Lindenkamp, Michel R. Osterhage, Cornelius Knabbe and Doris Hendig

Herz- und Diabeteszentrum Nordrhein-Westfalen, Institut für Laboratoriums- und Transfusionsmedizin, Universitätsklinik der Ruhr-Universität Bochum, Georgstraße 11, 32545 Bad Oeynhausen, Germany; swaldmann@hdz-nrw.de (S.J.); clindenkamp@hdz-nrw.de (C.L.); mosterhage@hdz-nrw.de (M.R.O.); cknabbe@hdz-nrw.de (C.K.); dhendig@hdz-nrw.de (D.H.)

* Correspondence: rpluemers@hdz-nrw.de

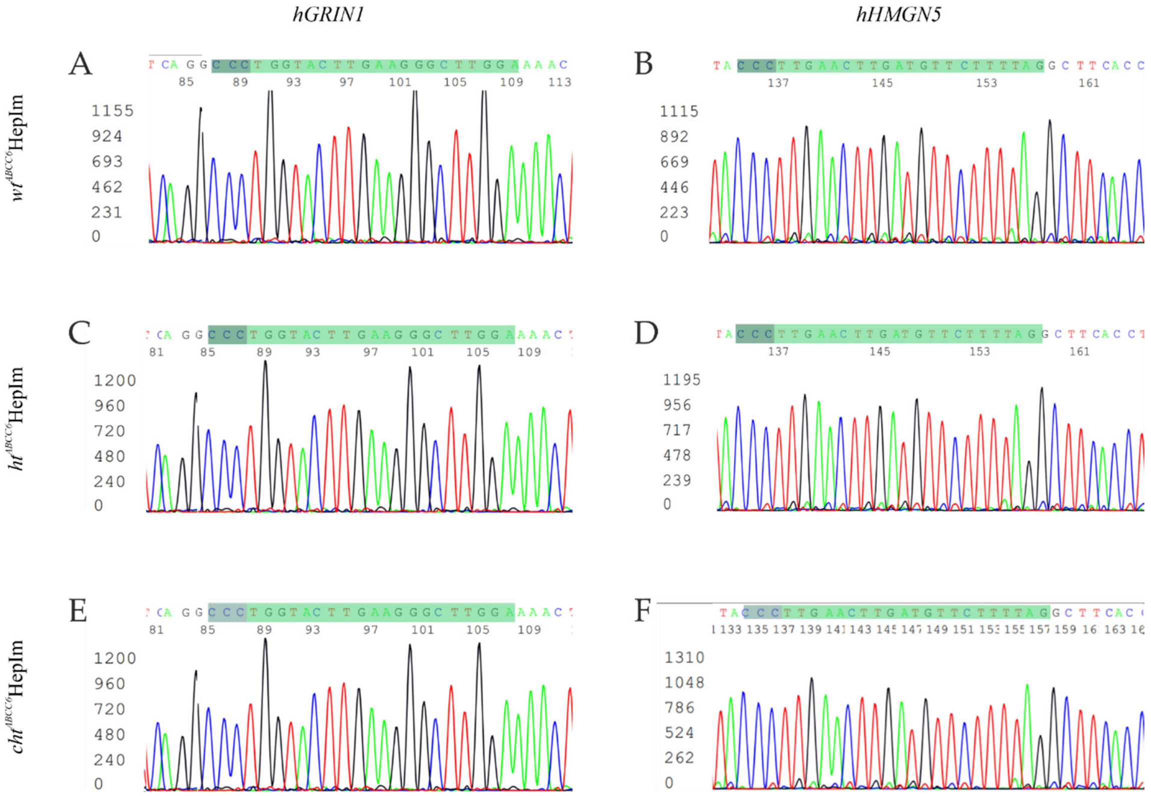

Figure S1. Sanger sequencing of the two most likely off targets. Off target analysis was performed in (**A**,**B**) *wt*^{ABCC6}HepIm, (**C**,**D**) *ht*^{ABCC6}HepIm and (**E**,**F**) *cht*^{ABCC6}HepIm after CRISPR-Cas9-mediated genome editing. The potential binding sites of the gRNA in (**A**,**C**,**E**) *hGRIN1* and (**B**,**D**,**F**) *hHMGN5* are marked in green.

 https://doi.org/10.3390/cells14080576
